# *Exocelina baliem* sp. n., the only known pond species of New Guinea *Exocelina* Broun, 1886 (Coleoptera, Dytiscidae, Copelatinae)

**DOI:** 10.3897/zookeys.304.4852

**Published:** 2013-05-28

**Authors:** Helena V. Shaverdo, Lars Hendrich, Michael Balke

**Affiliations:** 1 Naturhistorisches Museum, Burgring 7, A-1010 Vienna, Austria; 2 Zoologische Staatssammlung München, Münchhausenstraße 21, D-81247 Munich, Germany; 3 Zoologische Staatssammlung München, Münchhausenstraße 21, D-81247 Munich, Germany and GeoBioCenter, Ludwig-Maximilians-University, Munich, Germany

**Keywords:** *Exocelina*, diving beetles, Australasia, New Guinea, New Caledonia, taxonomy, new species, biogeography, phylogeny

## Abstract

*Exocelina baliem* **sp. n.** is described from the Baliem Valley in the Central Mountain Range of New Guinea (Papua Province, Indonesia). Having striolate elytra, different structure and setation of the male and female genitalia and tarsomeres, and inhabiting swampy ponds, the new species differs from all known New Guinea species, which have smooth elytra and are stream associated. It forms a monophyletic group with the Australian *Exocelina ferruginea* (Sharp, 1882) and New Caledonian *Exocelina inexspectata* Wewalka, Balke & Hendrich, 2010, based on shape of the paramere and structure of the male tarsi. Habitus, protarsomeres, and male and female genitalia are illustrated, comparing some structures with *Exocelina ferruginea* and two New Guinea stream species. We briefly discuss the biogeographic relevance of this discovery.

## Introduction

The Australasian Copelatinae genus *Exocelina* Broun, 1886 (= *Papuadytes* Balke, 1998, see Nilsson and Fery 2006 and Nilsson 2007) is a current taxonomic and molecular phylogenetic research focus (Balke and Bergsten 2003; Balke et al. 2004a, 2004b, 2007; Watts & Humphreys 2009; Wewalka et al. 2010). Here, we continue our study of the highly diverse New Guinea *Exocelina* fauna, with 63 formally described species (Balke 1998, 1999, 2001; Shaverdo et al. 2005, 2012). These species form a morphologically homogenous, monophyletic group associated with streams and rivers (e.g. Balke et al. 2007). In the present paper, we describe a new species, which is morphologically distinct and ecologically different from all other known New Guinea species. The closest related species to the new one occur in Australia and New Caledonia.

## Material and methods

Studied specimens are in the following collections:

**CLH** collection of Lars Hendrich, Munich, Germany (property of NHMW)

**NHMW** Naturhistorisches Museum Wien, Vienna, Austria (M.A. Jäch)

**MNHN** Muséum National d’Histoire Naturelle, Paris, France (T. Deuve, A. Mantilleri)

**ZSM** Zoologische Staatssammlung München, Munich, Germany (M. Balke)

Specimens of the following *Exocelina* species were studied for comparative purposes:

*Exocelina australiae* (Clark, 1863): 1 male, 1 female “Collect. Plason”, “Copelatus nigritulus Shrp. N. H.[?]” [hand written] (NHMW).

*Exocelina ferruginea* (Sharp, 1882): 1 male, 1 female “Australia / SA: Adelaide Hills, 5 km W Forreston, Mt. Crawford State Forest, Watts Gully, 200m, 22.11.1999, Hendrich & Watts leg. (Loc. 2/140)” (CLH).

*Exocelina inexspectata* Wewalka, Balke & Hendrich, 2010: holotype (male) “New Caledonia 12286, 20°25'S, 164°13'E, Nehoue campground, 29 Apr 2005, 10m G.B. Monteith, MV light.”, “HOLOTYPUS Exocelina inexpectata sp.n. Wewalka et al. 2008” [red, printed] (MNHN).

*Exocelina knoepfchen* Shaverdo, Hendrich, Balke, 2012: 1 male “Papua New Guinea: Eastern Highlands, Kainantu, Yoginofi, 1900m, 9.v.1994, 06.21.799S, 145.45.463E, Balke & Sagata (PNG 55)” (ZSM).

*Exocelina simplex* (Clark, 1863): 2 males, 2 females “South AU, nr. Penola, roadside pools, 37.380928°, 140.837540°, 30–31.10.2001, M. Balke leg.” (NHMW).

*Exocelina ullrichi* (Balke, 1998): 1 male “Papua New Guinea: Eastern Highlands, Hogu, 1 km E Mt. Barola, 1900m, 9.v.2006, 06.17.556S, 145.45.036E, Balke & Sagata (PNG 56)”(ZSM). 1 female “Papua New Guinea: Aiyura, 1787m, 15.i.2003, 06 21 411S, 145 54.340E, K. Sagata (WB5)” (ZSM).

All specimen data are quoted as they appear on the labels attached to the specimens. Label text is cited using quotation marks. Our red identification labels were attached to the types.

Measurements were taken using a Leica M205C stereomicroscope. The following abbreviations were used: TL (total body length), TL-H (total body length without head), and MW (maximum body width). Drawings were made with the aid of a *camera lucida* attached to a Leica DM 2500 microscope. For detailed study and illustration, protarsi and genitalia were removed and mounted on glass slides with DMHF (dimethyl hydantoin formaldehyde) as temporary preparations. The drawings were scanned and edited, using the software Adobe Illustrator CS5.1.

The terminology to denote the orientation of the genitalia (“ventral” for median lobe and gonocoxae and “dorsal” and “external” for paramere) follows Miller & Nilsson (2003). Administrative divisions of Indonesia follow information from Wikipedia (2013).

## Species description

### Exocelina baliem sp. n.

http://species-id.net/wiki/Exocelina_baliem

urn:lsid:zoobank.org:act:E1B1BD92-F118-4E18-AE45-62FD23EB7A68

Figs 1 –7

#### Type locality.

Indonesia: Papua Province: Jayawijaya Regency, Baliem River Valley, Wamena, 138°56'E, 04°06'S.

#### Type material.

*Holotype*: male “IRIAN JAYA Baliem-Tal Wamena, 1700 m 138°56'E, 04°06'S”, “20–27.9.1992 (54A = 57) leg. M. Balke” (NHMW). *Paratypes*: 2 males, 3 females with the same label as the holotype, 1 female additionally with two green labels “DNA”, “M.Balke 3268” (NHMW, ZSM). 1 male “W.-Neuguinea/Baliem Valley Wamena (Ort), 1600m / IR 1&6 31.8 & 6.9.1990 leg: Balke & Hendrich”, “Coll. Hendrich Berlin” (CLH). 1 female “IRIAN JAYA, Jayawijaya-Prov., leg. A.Riedel, 1993”, “Wamena, Baliem-River, 1700m, 15.X.” (ZSM).

#### Diagnosis.

Beetle middle-sized, piceous, with reddish brown head; both sexes matt, dorsal surface with strong dorsal microreticulation and numerous, short strioles; male antennomeres simple; male pro- and mesotarsomeres 1–3 distinctly dilated, male protarsomere 4 modified, with large, thick anterolateral hook-like seta, male protarsomere 5 simple, with relatively long setae and long claws, anterior claw with fine serration ventrally; median lobe with continuous outline in ventral view, ventral sclerite with a strip of sclerotization on right side, proximal part of median lobe striolate; paramere without notch on dorsal side but with a long prolongation of subdistal part; female metatarsi without ventral row of natatorial setae; gonocoxae with prolonged, slightly pointed apices. This is the only New Guinea species of *Exocelina* with a striolate dorsal surface.

#### Description.

**Figure 1.**
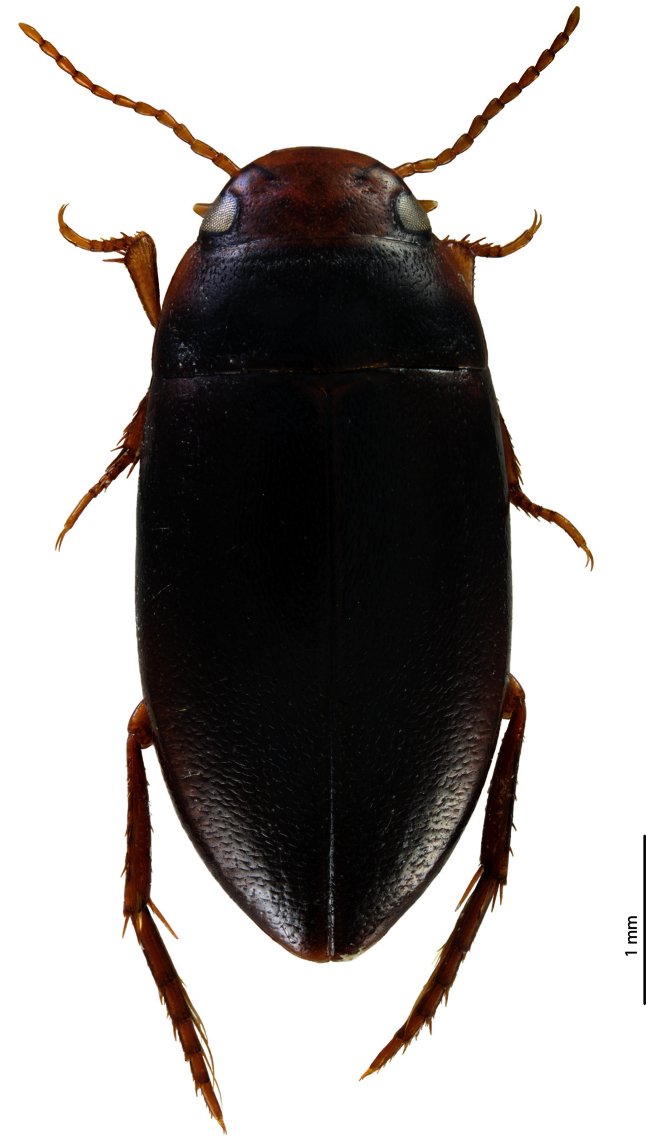
Habitus of *Exocelina baliem* sp. n., female.

*Size and shape*: Beetle middle-sized (TL-H 4.2–4.5 mm, TL 4.7–5.1 mm, MW 2.2–2.3 mm), one female larger (TL-H 4.9 mm, TL 5.5 mm, MW 2.4 mm), with elongate habitus, broadest at elytral middle; pronotum relatively long (width of pronotum/length of pronotum ratio 0.4), only slightly trapezoidal, with sides weakly converging forwards, with posterior angles not drawn backwards (Fig. 1). *Coloration*: Head reddish brown, with darker, indistinct, broad, V-shaped median spot and dark brown posteriorly to eyes; pronotum piceous, with anterior margin and anterior angles reddish brown to brown; elytra piceous, sometimes with paler (reddish brown to dark brown) posterolateral sides, apex, and narrow bands along elytral suture; head appendages yellowish to reddish-brown, hind legs darker; ventrally reddish brown, with piceous metaventrite and metacoxal plates.

*Surface sculpture*: Head with dense, coarse punctures (spaces between punctures 1–3 times size of punctures, diameter of punctures much larger than diameter of cells of microreticulation) on middle, anterior part of head with finer punctation, between and behind eyes with very short but distinct longitudinal strioles, vertex with fine, sparse punctation. Pronotum with numerous short longitudinal strioles, distinctly shorter and sparser on disc, disc also with coarse punctures. Elytra densely covered with numerous short longitudinal strioles, posterior third of elytra with transverse shackle-like strioles, and elytral lateral margins with transverse strioles and coarse punctures. Head, pronotum, and elytra with strongly impressed microreticulation, dorsal surface matt. Metaventrite and metacoxa distinctly microreticulate. Metacoxal plates densely covered with short longitudinal strioles and in anterior part also with transverse wrinkles. Abdominal ventrites with finer microreticulation and fine sparse punctation, more evident at their middle. Ventrites 1–2 with numerous longitudinal striae, ventrites 3–5 with finer, shorter, transverse strioles, and ventrite 6 with long sublongitudinal strioles.

*Structures*: Pronotum with distinct lateral bead. Base of proste rnum and neck of prosternal process with distinct ridge, without anterolateral extensions. Blade of prosternal process lanceolate, relatively broad, convex, with distinct bead and very few fine setae; neck and blade of prosternal process evenly jointed. Abdominal ventrite 6 broadly rounded apically.

**Figure 2.**
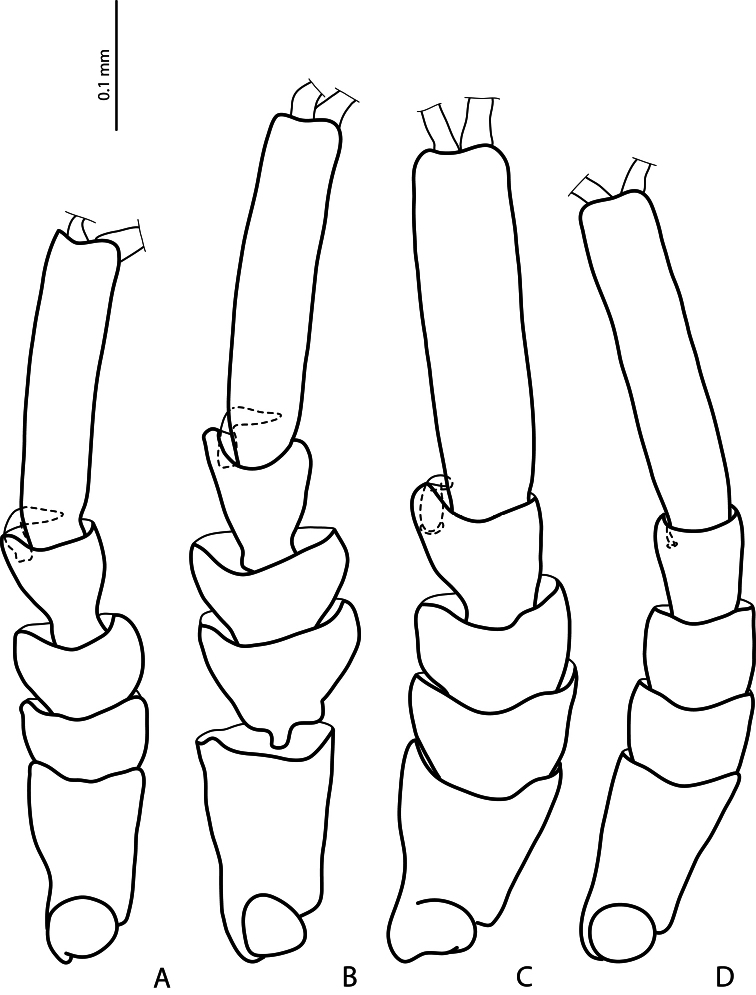
Male protarsomeres 1–5 in dorsal view **A** *Exocelina baliem* sp. n. **B** *Exocelina ferruginea* (Sharp, 1882) **C** *Exocelina ullrichi* (Balke, 1998) **D** *Exocelina knoepfchen* Shaverdo, Hendrich, Balke, 2012.

**Figure 3.**
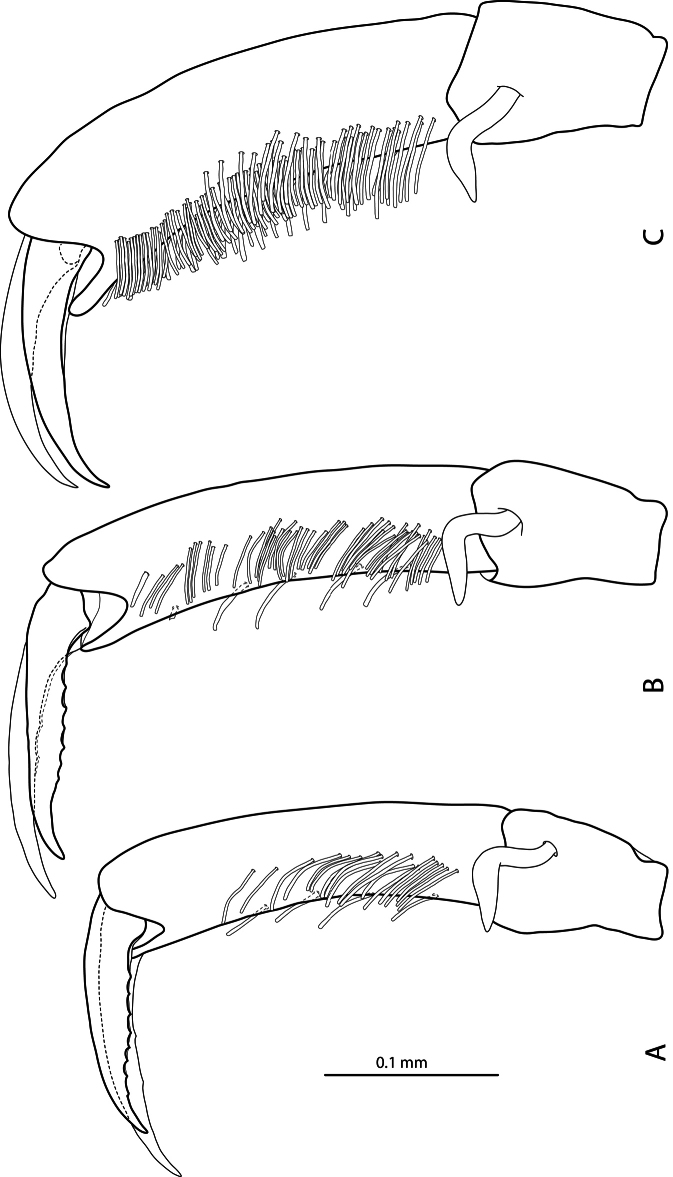
Male protarsomeres 4–5 in lateral view **A** *Exocelina baliem* sp. n. **B** *Exocelina ferruginea* (Sharp, 1882) **C** *Exocelina ullrichi* (Balke, 1998).

**Figure 4.**
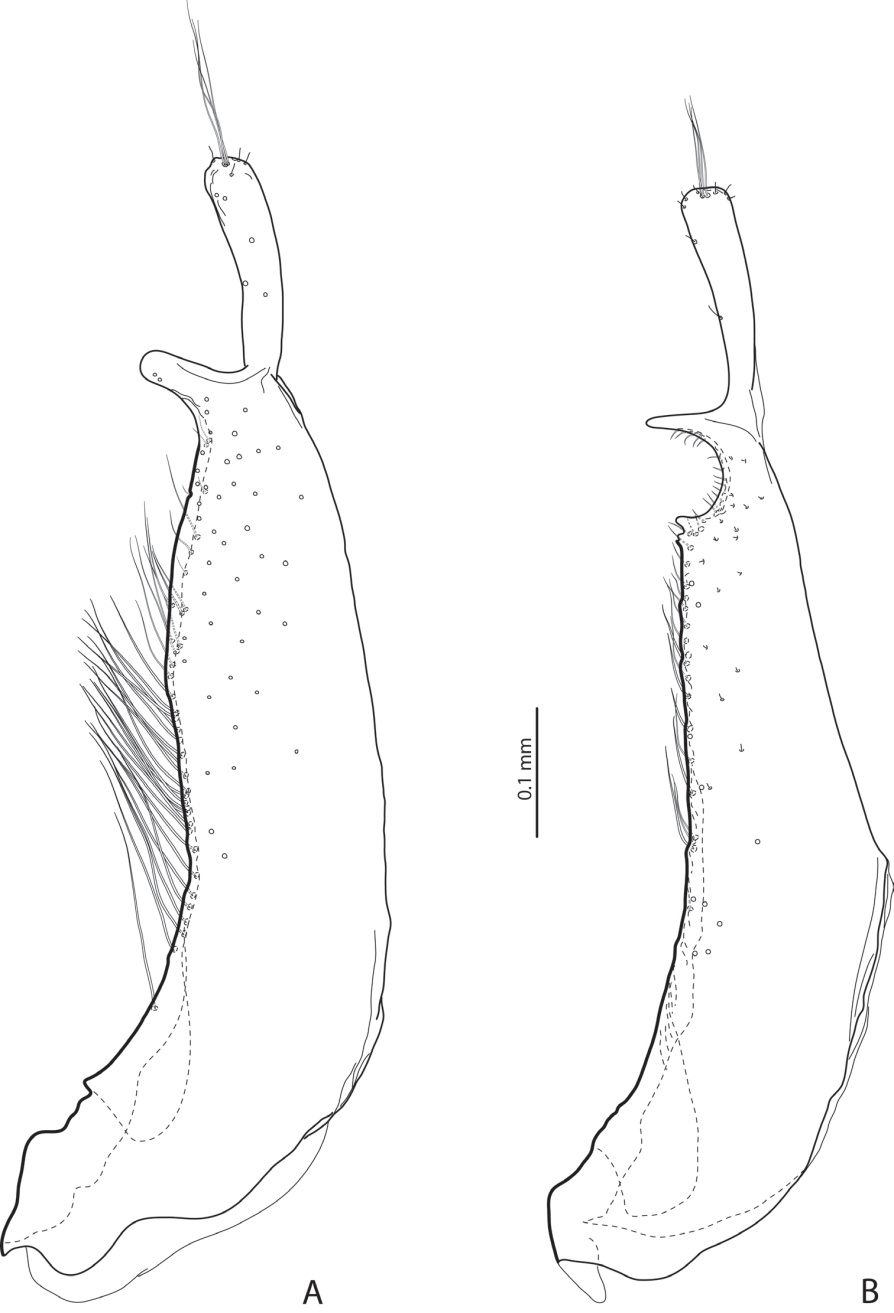
Paramere in external view **A** *Exocelina baliem* sp. n. **B** *Exocelina ferruginea* (Sharp, 1882).

**Figure 5.**
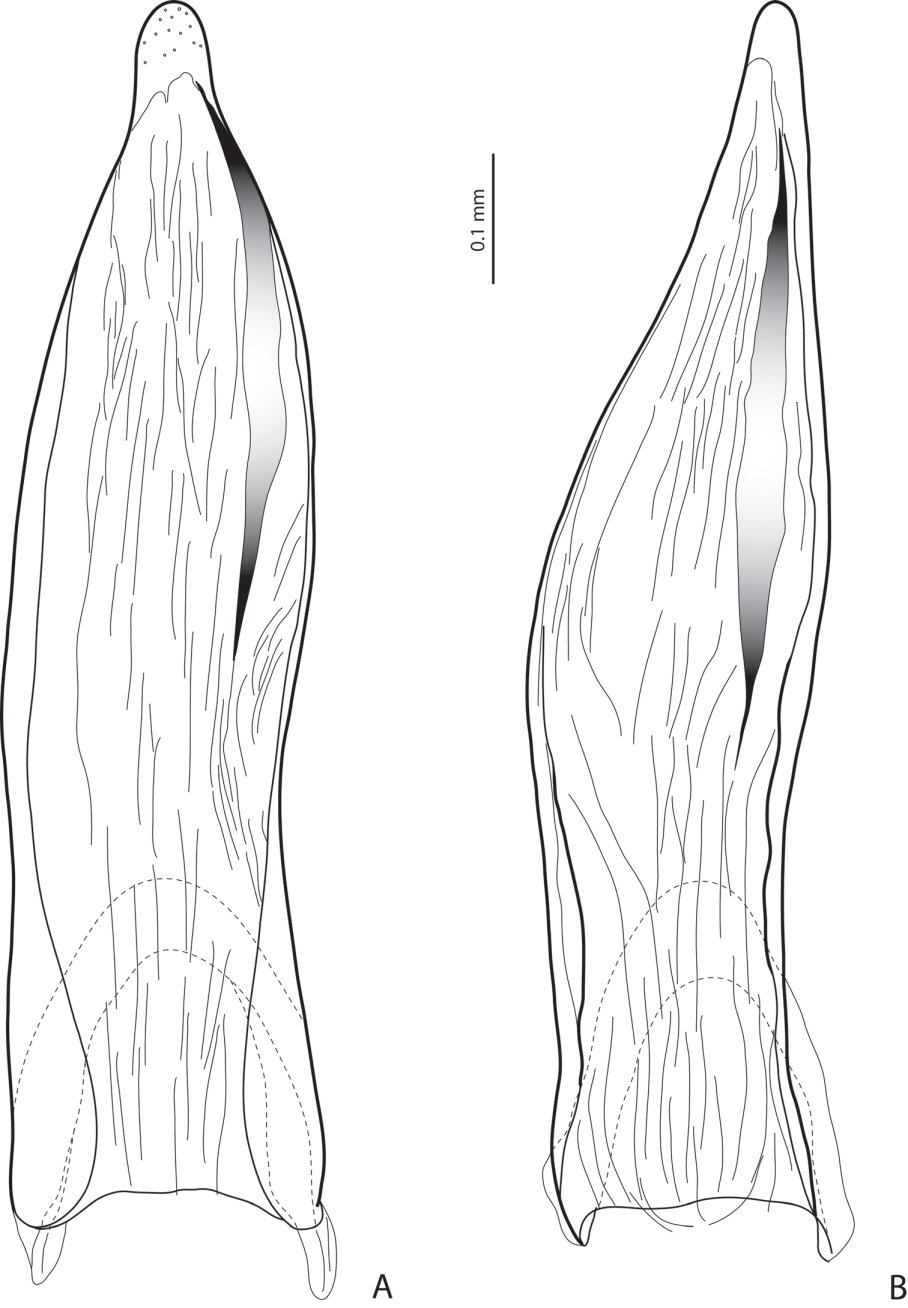
Median lobe in ventral view **A** *Exocelina baliem* sp. n. **B** *Exocelina ferruginea* (Sharp, 1882).

**Figure 6.**
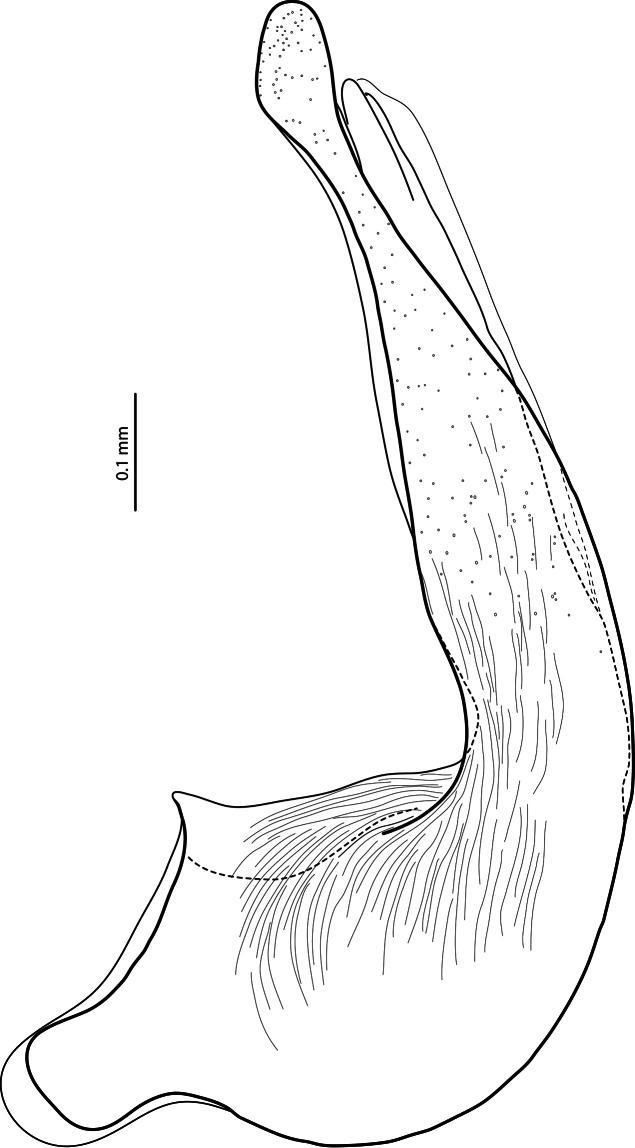
Median lobe in lateral view of *Exocelina baliem* sp. n.

*Male*: Antennomeres simple. Pro- and mesotarsomeres 1–3 distinctly dilated. Protarsomere 4 asymmetrical, its anterior angle expanded with large, thick, strongly curved anterolateral hook-like seta. Protarsomere 5 simple, ventrally with anterior row of 17–18 and posterior row of 4 long setae; pro- and mesotarsal claws long (length of anterior claw/length of protarsus ratio 0.7), posterior protarsal claw evenly curved, with two fine denticles on ventral margin; anterior claw longer, straighter, and slightly broadened, with fine serration ventrally (Figs 2A, 3A). Abdominal ventrite 6 with 20–25 lateral striae on each side. Median lobe with continuous outline, slightly asymmetrical in ventral view; apex of median lobe swollen in lateral view and roundly pointed in ventral view, ventral sclerite with a strip of sclerotization on right side in ventral view, proximal part of median lobe striolate (Figs 5A, 6). Paramere without notch (for comparison, e.g. see Figs 1–4 in Shaverdo et al. 2012) but with a long prolongation on subdistal part of dorsal side (Fig. 4A).

**Figure 7.**
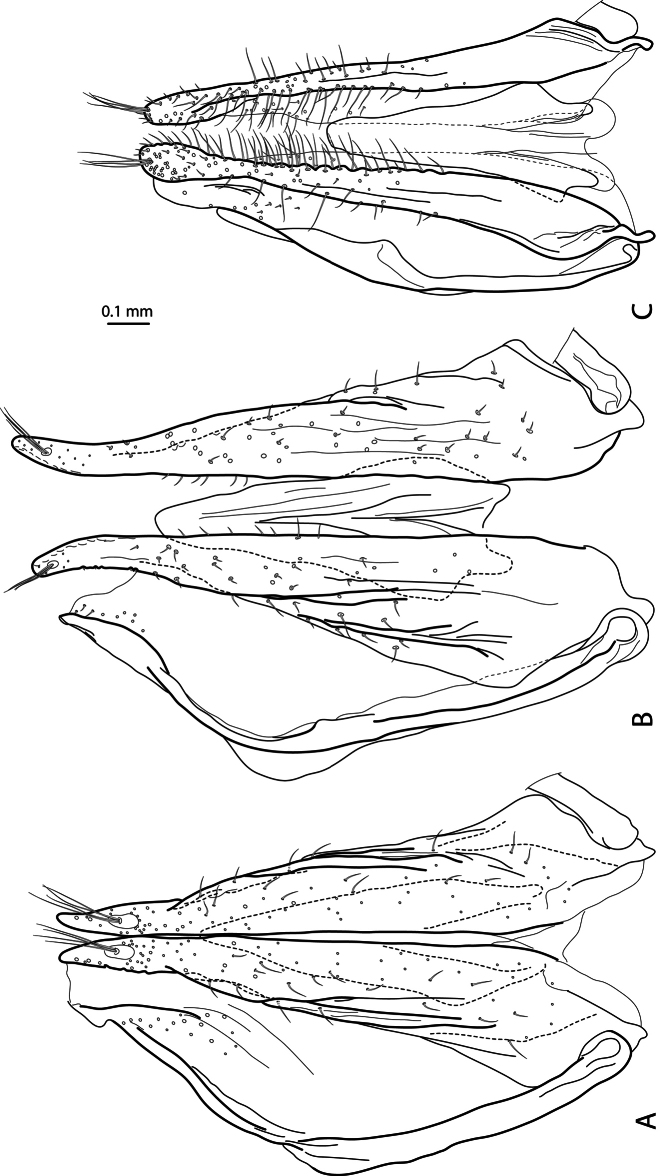
Female genitalia: gonocoxae, laterotergite, and rami in ventral view **A** *Exocelina baliem* sp. n. **B** *Exocelina ferruginea* (Sharp, 1882) **C** *Exocelina ullrichi* (Balke, 1998).

*Female*: Dorsal microreticulation stronger, abdominal ventrite 6 without or with 1–2 very fine median striae. Metatarsi without ventral row of natatorial setae. Gonocoxosternites similar to those of *Exocelina vladimiri* Shaverdo, Sagata & Balke, 2005 (see Fig. 17a in Shaverdo et al. 2005). Gonocoxae with prolonged, slightly pointed apices and sparse setation, without setae on inner margin in ventral view (Fig. 7A).

#### Distribution and habitat.

**Figure 8.**
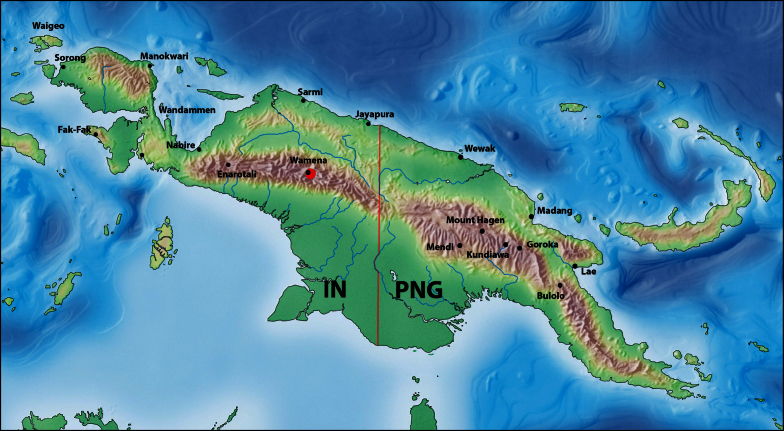
Map of New Guinea showing the type locality (red dot) of *Exocelina baliem* sp. n.

The species is known only from the type locality (Fig. 8).

**Figure 9.**
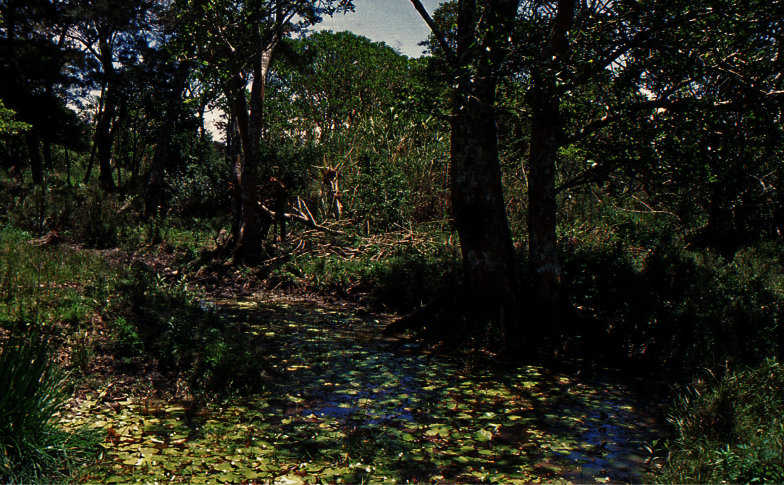
Type locality of *Exocelina baliem* sp. n.

The species was collected from a small pool in a riverine relic forest close to the Baliem River, approximately 1 km from the runway of Wamena airport (Fig. 9). The beetles were found between roots, leaves, and emergent water plants in the shallow water, at the shady edge of the pond underneath a large tree. One specimen was collected from a tuft of *Phragmites* after the pool had dried up during the rather dry summer in the following year (1993). The new species was associated with the following dytiscids: *Hydrovatus enigmaticus* Biström, 1997, *Hyphydrus dani* Biström, Balke & Hendrich, 1993, *Hydaticus okalehubyi* Balke & Hendrich, 1992, and *Rhantus dani* Balke, 2001. We revisited the area in winter 2011 and found that most ponds were highly eutrophic (as foreseen by Balke 1993), large trees had mostly disappeared, and the species was not found again during a quick survey. We assume that the type locality has been destroyed, but other suitable habitats might exist elsewhere in the vast valley.

#### Etymology.

The species is named after the type locality, the Baliem River Valley. The name is a noun in the nominative singular standing in apposition.

## Comparison and discussion

The new species is morphologically similar to the Australian *Exocelina ferruginea* (Sharp, 1882) and the New Caledonian *Exocelina inexspectata* Wewalka, Balke & Hendrich, 2010. It shares with them some characters not found in all other known New Guinea *Exocelina* and listed below.

1) Striolate elytra (Fig. 1); in *Exocelina ferruginea* and *Exocelina inexspectata* elytra without strioles on anterior (basal) 2/3 but with distinct punctures, and in posterior (apical) third with transverse strioles. The striolate dorsal surface of the body is also characteristic for other Australian and New Caledonian species. All other New Guinea species have punctation rather than strioles on elytra.

2) Modification of paramere: prolongation of subdistal part on dorsal side (Figs 4A, 4B). This apomorphic character is observed only in these three species.

3) Presence of a strip of sclerotization on right side of ventral sclerite of median lobe (Figs 5A, 5B). This character is also present in *Exocelina ferruginea*. The holotype of *Exocelina inexspectata* is teneral so this character is not evident, but is characteristic for several other New Caledonian species, e.g. *Exocelina novaecaledoniae* (J. Balfour-Browne, 1939), *Exocelina ouin* Wewalka, Balke & Hendrich, 2010, and *Exocelina jeannae* Wewalka, Balke & Hendrich, 2010.

4) Striolate surface of proximal part of median lobe (Fig. 6). It is characteristic of Australian species.

5) Similar modification of pro- and mesotarsus (Figs 2, 3):

- Pro- and mesotarsomeres 1–3 distinctly dilated.

- Protarsomere 4 asymmetrical, with expanded anterior angle where there is a large, strongly curved anterolateral hook-like seta.

*Exocelina ullrichi* (Balke, 1998) is the only other species from New Guinea, which shares these two characters, see Fig. 29 in Balke (1998).

- Protarsomere 5 simple, ventrally with anterior row of numerous and posterior row of very few long setae. This character is also found in *Exocelina ferruginea* and in *Exocelina inexspectata*.

- Pro- and mesotarsal claws long.

- Anterior claw longer than posterior, straighter, and slightly broadened.

The above mentioned modifications of pro- and mesotarsus are characteristic of all known Australian species, with some small variations.

- Anterior claw with fine serration ventrally. This character is also observed in *Exocelina ferruginea* and *Exocelina inexspectata*.

- Posterior protarsal claw evenly curved, with two fine denticles on ventral margin. This character is also present in *Exocelina ferruginea*.

6) Female metatarsi without ventral row of natatorial setae. It is characteristic of known Australian species (in *Exocelina australiae* (Clark, 1863) also for males).

7) Shape and setation of gonocoxae: apices not rounded, slightly pointed, setation much sparser (Fig. 7A). The gonocoxae of *Exocelina baliem* are evidently different from those of other New Guinea *Exocelina* species (Fig. 7C and Fig. 17b in Shaverdo et al. 2005) and much more similar to the gonocoxae of *Exocelina ferruginea* (Fig. 7B).

*Exocelina baliem* sp. n. is unique among known New Guinea *Exocelina* species in its habitat requirements. It inhabits ponds, unlike all other known species of *Exocelina* in New Guinea, which occur in directly stream related stagnant water, such as rockpools, stagnant backflows, marginal puddles and waterholes along stream banks, and at the immediate stream margin. The habitat of *Exocelina baliem* sp. n. is similar to that of the closely related *Exocelina ferruginea*.

Balke et al. (2007) showed that New Guinea *Exocelina* species form a monophyle-tic group, the result of a single colonization from Australia, and that Australian species, including *Exocelina ferruginea*, form a "basal" paraphyletic series, followed by the other *Exocelina* species. Thus, being closely related to *Exocelina ferruginea*, *Exocelina baliem* sp. n. represents an older colonization from Australia into New Guinea, but without subsequent radiation. This radiation likely occurred more recently, producing the highly diverse clade of morphologically distinct stream species. Also we assume that New Caledonia was colonized three times out of Australia, not twice as suggested by Balke et al. (2007), since the New Caledonian *Exocelina inexspectata* is closely related to *Exocelina baliem* sp. n. and *Exocelina ferruginea*. The species is known only from the holotype collected at light, and it might also be a pond species, whereas all other known New Caledonian species are stream associated.

## Supplementary Material

XML Treatment for Exocelina baliem
